# Patient-Specific Timing for Pulmonary CT Angiography: Human Validation of a Physiologically Derived Post-Trigger Delay Model

**DOI:** 10.3390/diagnostics16183033

**Published:** 2026-09-19

**Authors:** Kyrillos Grace, Neda Ziaei, Lynn Duong, Alireza Shojazadeh, Liqiang Ren, Cecilia Brewington, Sabee Molloi

**Affiliations:** 1 Department of Radiological Sciences, University of California, Irvine, CA 92697, USA; kyrillog@hs.uci.edu (K.G.); ziaein@uci.edu (N.Z.); lynnqd@uci.edu (L.D.); ashojaza@hs.uci.edu (A.S.); 2 Department of Radiology, University of Texas Southwestern Medical Center, Dallas, TX 75390, USA; liqiang.ren@utsouthwestern.edu (L.R.); cecelia.brewington@ochsner.org (C.B.)

**Keywords:** computed tomography angiography, contrast timing, post-trigger delay, bolus tracking

## Abstract

**Objective:** To evaluate the ability of a physiologically derived post-trigger delay (PTD) model to predict pulmonary arterial peak enhancement timing using human dynamic CT perfusion data and to compare physiologically derived PTD estimates with commonly used fixed-delay protocols. **Methods:** In this retrospective study, 39 patients who underwent dynamic pulmonary CT perfusion imaging were evaluated. Pulmonary arterial enhancement curves were generated from the main pulmonary artery (MPA) using attenuation measurements obtained from 19 dynamic CT volume scans and fitted using a gamma-variate function. Reference peak enhancement timing was determined from the fitted enhancement curves. A physiologically derived PTD was calculated using a previously established relationship incorporating contrast injection duration and a dispersion component derived from first-pass contrast transport dynamics. Fixed PTD protocols of 4, 5, 6, and 7 s were evaluated as reference benchmarks. Enhancement and contrast-to-noise ratio (CNR) measurements were obtained in the MPA and five lobar pulmonary arteries. Protocol performance was assessed relative to the reference standard using nonparametric statistical testing, regression analysis, and Bland–Altman analysis, with enhancement and CNR measurements summarized as median [interquartile range] and paired differences reported with 95% confidence intervals. **Results:** The mean physiologically derived PTD was 5.06 s, compared with a mean reference PTD of 5.18 s. In the MPA, the median absolute enhancement difference relative to the reference was 1.02 HU (95% CI, 0.42–4.66) for the physiologically derived PTD, compared with 50.76 HU (35.53–66.88), 1.05 HU (0.48–8.35), 25.60 HU (17.95–40.34), and 114.66 HU (78.45–138.55) for the 4-, 5-, 6-, and 7-s fixed-delay protocols, respectively. The corresponding median absolute CNR differences were 0.02 (0.01–0.10), 0.89 (0.67–1.23), 0.02 (0.01–0.16), 0.51 (0.32–0.78), and 1.96 (1.46–2.67). The physiologically derived PTD demonstrated significantly smaller differences from the reference than the 4-, 6-, and 7-s protocols (Holm-adjusted *p* < 0.05), with no significant difference compared with the 5-s protocol. Regression and Bland–Altman analyses demonstrated strong agreement with the reference standard. **Conclusions:** A physiologically derived PTD demonstrated strong agreement with retrospectively determined pulmonary arterial peak enhancement timing in this human cohort. While performance was similar to a fixed 5-s delay under the relatively standardized injection conditions used in this study, the findings support the applicability of a previously established physiologic PTD relationship to human pulmonary arterial enhancement and provide validation of the model using human dynamic CT data.

## 1. Introduction

Computed tomography pulmonary angiography (CTPA) is widely used for evaluation of the pulmonary arterial circulation due to its high spatial resolution, rapid acquisition, and broad clinical availability [1,2,3]. Despite substantial advances in multidetector CT technology, achieving consistent pulmonary arterial enhancement remains an important technical challenge because image quality depends on accurate synchronization between image acquisition and the passage of contrast material through the pulmonary vasculature [4,5,6,7]. Variability in contrast enhancement can arise from differences in contrast administration as well as underlying physiologic factors that influence first-pass contrast transport.

Pulmonary arterial enhancement is affected by multiple technical and physiologic variables, including contrast injection rate, injection duration, cardiac output, circulating blood volume, and patient body habitus [4,6,8,9,10,11,12,13]. Specifically, higher iodine flow rates significantly improve pulmonary artery enhancement, while cardiac output is a primary determinant of both the magnitude and timing of arterial enhancement [14,15,16,17]. Body weight and age have been identified as the only consistent independent predictors of pulmonary artery enhancement in clinical cohorts [8]. These factors influence both the magnitude and temporal distribution of contrast enhancement within the pulmonary circulation, resulting in variability in the timing of peak enhancement among patients [15,18,19]. To compensate for this variability, most CTPA protocols employ either test bolus techniques or bolus-tracking methods to determine scan timing [20].

The test bolus technique estimates scan timing using a preliminary low-volume contrast injection and assumes that the enhancement characteristics of the test bolus accurately predict those of the subsequent diagnostic injection [21,22]. However, differences in injection duration, bolus geometry, and physiologic conditions between the test and diagnostic injections may influence contrast enhancement dynamics and affect the accuracy of timing predictions [10,12,21]. Bolus tracking avoids the need for a preliminary injection by continuously monitoring attenuation within a target vessel and initiating image acquisition once a predefined attenuation threshold is reached [6,21]. Although this approach accounts for differences in contrast arrival time to the test bolus location primarily due to cardiac output and circulating blood volume, image acquisition is typically delayed by a fixed post-trigger interval. Consequently, the relationship between trigger detection and peak enhancement remains dependent on the contrast injection protocol [4,6,10,12,23].

Previous investigations of first-pass contrast transport have demonstrated that the temporal width of a contrast bolus is strongly related to injection duration and that, for relatively short contrast injection durations, peak enhancement occurs near the temporal center of the dispersed bolus [24,25]. Building upon these observations, a physiologically derived post-trigger delay (PTD) model was developed from dynamic contrast-enhanced CT studies and subsequently evaluated under controlled experimental conditions in swine models with varying cardiac outputs and injection protocols [24,25,26]. These investigations demonstrated that the optimal PTD following contrast triggering could be approximated by one-half of the contrast injection duration plus a dispersion component accounting for transport and mixing of contrast material within the circulation [24,25,26]. The dispersion component was not intended to represent a specific physiologic variable, but rather a lumped description of the residual transport and mixing effects that remain after triggering and are not directly captured by bolus duration alone. Across a range of experimental conditions, the resulting model demonstrated robust agreement with observed peak enhancement timing, suggesting that it captures fundamental characteristics of first-pass contrast transport rather than reflecting a single experimental condition [24,25,26].

The PTD model evaluated in the present study was previously developed from experimental first-pass contrast transport data and was not derived from the current dataset [24,25,26]. Its applicability to human pulmonary arterial enhancement, however, has not been fully evaluated. Therefore, the present investigation was designed to externally validate this established physiologic relationship using human dynamic CT data.

Although contrast injection protocols were relatively standardized within this cohort, individual enhancement curves remained subject to physiologic variability arising from differences in contrast transport and circulatory dynamics. Dynamic pulmonary CT perfusion imaging provides a unique opportunity to characterize these enhancement patterns by generating patient-specific enhancement curves and corresponding estimates of peak enhancement timing.

Therefore, the purpose of this study was to evaluate the ability of a physiologically derived PTD model to predict pulmonary arterial peak enhancement timing using dynamic human CT perfusion data. Model-derived PTD estimates were evaluated against peak enhancement timing obtained from gamma-variate analysis, while commonly used fixed PTD protocols were included as reference benchmarks for comparison.

## 2. Materials and Methods

### 2.1. Study Design and Patient Population

This retrospective study was approved by the institutional review board from UT Southwestern Medical Center, and all subjects provided written informed consent at the time of imaging. The study was conducted in accordance with the Declaration of Helsinki. Institutional review board approval was obtained from UT Southwestern Medical Center (Approval No: STU 122011-106; Date: 28 September 2012). A total of 48 patients were identified from the individuals who underwent pulmonary CT perfusion imaging for evaluation of suspected pulmonary lesions between December 2012 and August 2014.

**Figure 1:**
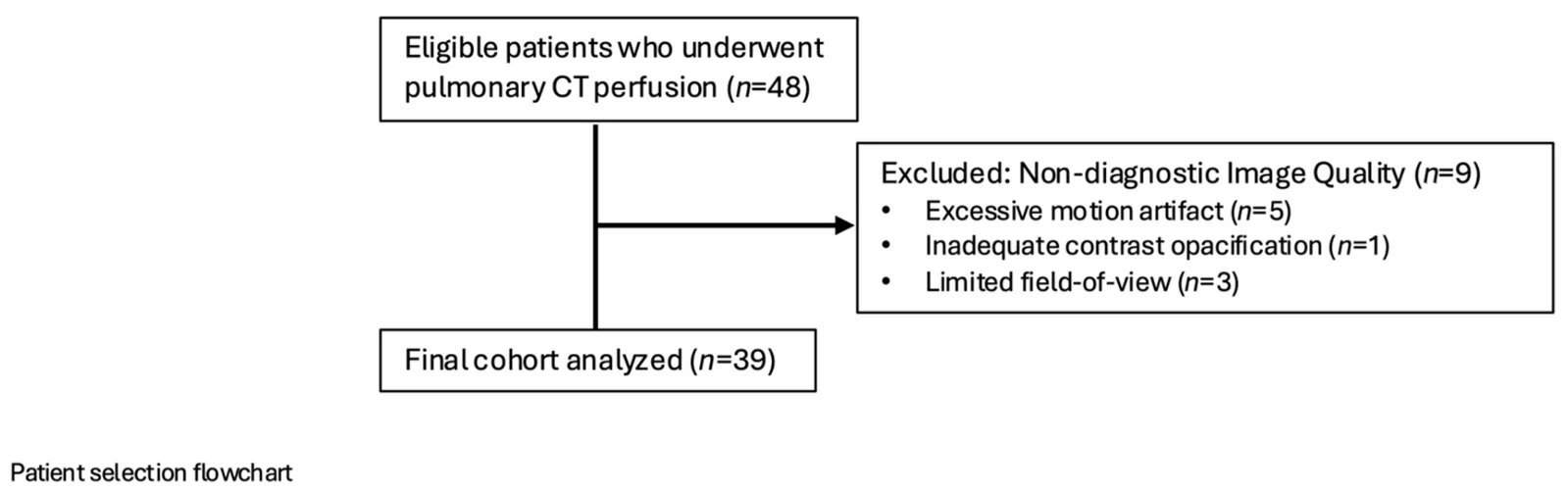
Patient selection flowchart: 48 eligible patients who underwent CT perfusion were identified; 9 patients were excluded due to nondiagnostic image quality, leaving 39 patients for final analysis.

Eligible subjects were adults (>18 years) with non-calcified pulmonary lesions (nodule or mass) measuring 6–30 mm in diameter who were capable of breath-holding for at least 20 s. Patients with known iodinated contrast allergy, severe renal impairment (estimated glomerular filtration rate < 50 mL/min or serum creatinine > 1.5 mg/dL), advanced emphysema, supplemental oxygen dependence, cardiomyopathy, pulmonary hypertension, or severe hypersensitivity to iodinated contrast media were excluded. Nine patients were subsequently excluded because of excessive motion artifact, inadequate contrast opacification, or field-of-view limitations, resulting in a final study cohort of 39 patients (Figure 1).

### 2.2. CT Imaging Protocol

All imaging examinations were performed on a 320-detector CT scanner (Aquilion ONE, Toshiba Medical Systems, Otawara, Japan) using dynamic area-detector coverage of 16 cm centered on the lesion or region of interest. Imaging parameters included detector collimation of 320 × 0.5 mm, tube voltage of 80 kVp, tube current of 120 mA, and a gantry rotation time of 0.5 s.

Images were reconstructed on a 512 × 512 matrix with a 350-mm field of view and 2-mm section thickness using AIDR 3D Standard iterative reconstruction (Toshiba Medical Systems, Otawara, Japan).

Intravenous contrast material (Isovue 370; 370 mg I/mL; Bracco Diagnostics, Princeton, NJ, USA) was administered at a volume of 40–50 mL at a rate of 5 mL/s using a dual-head power injector, corresponding to a contrast injection duration of 8–10 s. Contrast administration was followed by a 20-mL saline flush delivered at the same injection rate. Dynamic acquisitions were obtained every 2 s during the first 28 s following contrast administration and at additional delayed time points (40, 50, 60, 90, and 120 s), resulting in a total of 19 scan series per patient.

### 2.3. Radiation Dose Parameters

Each 0.5-s acquisition produced a volumetric CT dose index (CTDIvol) of approximately 2 mGy using the 80 kVp and 120 mA protocol, corresponding to a dose-length product (DLP) of approximately 32 mGy·cm per acquisition based on the 16-cm scan coverage. Across all 19 acquisitions, the cumulative DLP was approximately 608 mGy·cm, corresponding to an estimated effective radiation dose of approximately 8.5 mSv using an adult chest conversion factor (k) of 0.014 mSv·mGy^−1^·cm^−1^ [27].

### 2.4. Reference Pulmonary Arterial Enhancement Curves

The main pulmonary artery (MPA) served as the primary region of interest (ROI) for generation of pulmonary arterial enhancement curves. For each patient, attenuation measurements obtained from all 19 dynamic acquisitions were used to construct a first-pass contrast enhancement curve describing pulmonary arterial enhancement over time.

**Figure 2:**
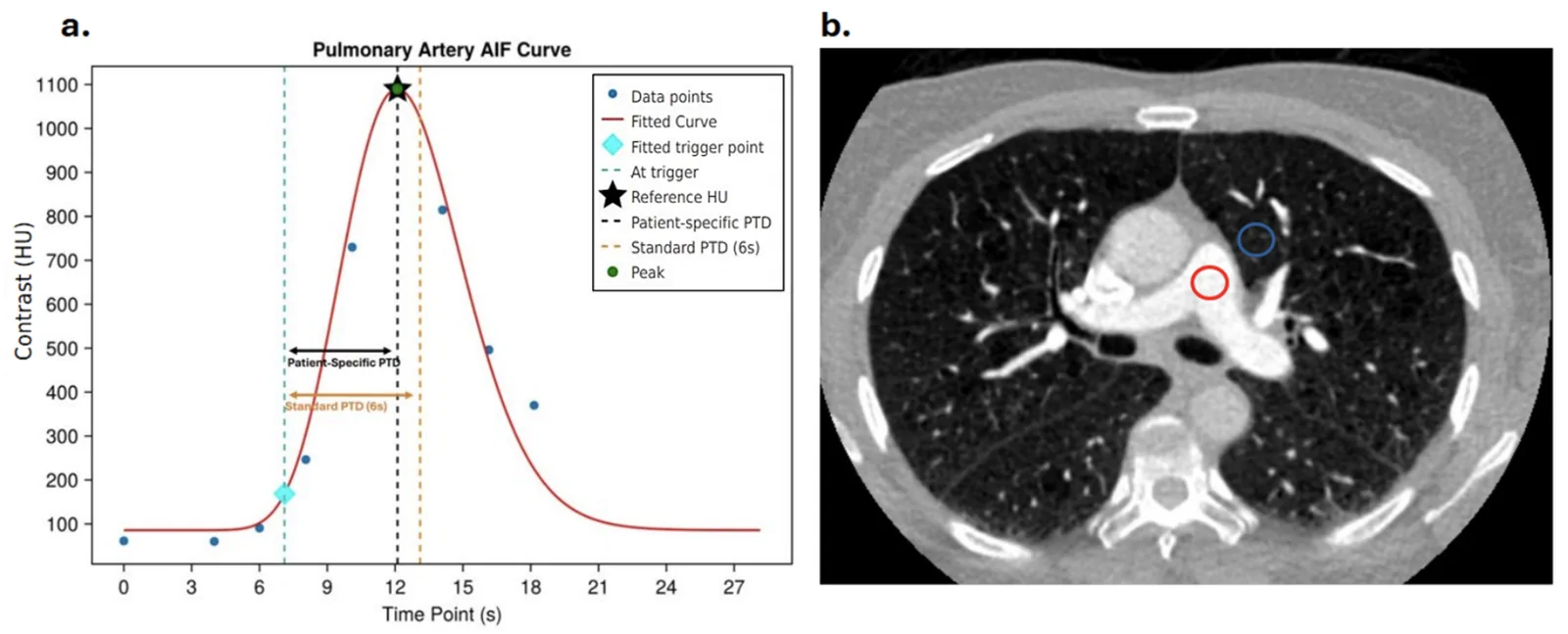
AIF curves for the MPA and the corresponding axial CTPA image. Image (**a**) shows the fitted time-intensity curve displaying contrast pass curve in the MPA with markers for trigger point, patient-specific PTD, 6-s standard PTD, and peak enhancement. Image (**b**) shows the ROI placement in the MPA (red circle) and in background air (blue circle). AIF: arterial input function; MPA: main pulmonary artery; CTPA: computed tomography pulmonary angiography; PTD: post-trigger delay; ROI: region of interest.

To reduce the influence of image noise and discrete sampling intervals, enhancement curves were fitted using a gamma-variate function previously described for modeling first-pass contrast kinetics [24,25]:

(1) $$\mathrm{Enhancement}\;\mathrm{Curve}=A\cdot {\left(\frac{t}{T}\right)}^{B}\cdot {exp}^{B\cdot (1-\frac{t}{T})}$$

where A is the peak enhancement, T is the TTP, B shapes the curve, C is the baseline HU, and t is time. In addition, gamma-variate fitting was performed using a fully automated procedure without manual adjustment of the fitted curves. The fitted enhancement curve was used to estimate the time corresponding to maximum pulmonary arterial enhancement. This calculated peak enhancement served as the reference standard for timing analysis. A simulated bolus-tracking trigger threshold of 150 HU was then applied to each fitted curve, and the interval between the trigger point and the fitted peak enhancement was defined as the reference PTD for that patient (Figure 2). The 150-HU threshold was selected based on its established use in pulmonary arterial bolus-tracking protocols and prior studies of contrast time-enhancement curves demonstrating favorable pulmonary arterial enhancement using a 150-HU trigger in combination with a PTD [28,29].

### 2.5. Physiologically Derived Post-Trigger Delay Model

A physiologically derived PTD model previously developed from dynamic contrast-enhanced CT studies was evaluated in this investigation [24,25,26]. The model was not derived from the current dataset. Rather, it was established from prior studies examining first-pass contrast transport dynamics and subsequently evaluated under controlled experimental conditions across varying injection protocols and hemodynamic states.

The model is based on the observation that, for relatively short contrast injections, first-pass enhancement curves approximate a near-symmetric distribution, with peak enhancement occurring near the temporal center of the dispersed contrast bolus. Accordingly, the optimal PTD can be estimated as one-half of the contrast injection duration plus a dispersion component accounting for residual transport and mixing effects within the circulation [25].

The physiologic PTD model was calculated as follows

(2) $$T_{p}=\alpha \cdot \frac{T_{inj}}{2}+D_{x}$$

where *Tp* is the post-trigger delay (PTD), *T_inj_* isthe total contrast injection duration, α is a scaling factor that adjusts for bolus shape, and *Dx* is a fixed dispersion delay. In this study, we implemented this model using the following empirically derived parameters based on prior studies [25,26].

(3) $$T_{p}\;=\;1.01\;\cdot \;\frac{T_{inj}}{2}\;+\;1.00$$

For each patient, the model-derived PTD was calculated using the administered injection duration. The resulting PTD estimate represented the predicted delay between attainment of the trigger threshold and peak pulmonary arterial enhancement.

### 2.6. Fixed-Delay Reference Protocols

To provide clinically relevant benchmarks, fixed PTD of 4, 5, 6, and 7 s were evaluated. These delay intervals were selected because they encompass commonly used post-trigger delays in clinical CTPA protocols.

The purpose of these comparisons was not to establish a reference standard but rather to provide context for interpretation of model-derived PTD performance relative to fixed-delay timing approaches currently used in practice.

### 2.7. Image Processing and Analysis

The MPA, proximal to its bifurcation, served as the primary ROI for bolus tracking, with additional ROIs placed immediately distal to the bifurcation in the five lobar branches corresponding to the left upper and lower lobar arteries (LUL, LLL), and the right upper, middle, and lower lobar arteries (RUL, RML, RLL). Vessel ROIs were sized to span the entire cross-sectional area of each artery, avoiding contact with the vessel wall to minimize partial volume effect from surrounding tissue, and excluding areas with predominant calcification. ROI placement and image analysis were independently performed by three trained observers (K.G., N.Z., L.D.), who were blinded to the calculated PTD values. Each observer independently placed all ROIs de novo, without copying or adjustment of ROIs placed by another observer.

For each scan, HU values were measured in the MPA and its lobar branches to quantify contrast enhancement. Contrast-to-noise ratio (CNR) was calculated using the mean contrast of the corresponding ROI and the standard deviation of the background, defined as a 10-mm circular ROI placed in adjacent lung parenchyma, which served as a consistent low-density reference (Equation (4)).

(4) $$CNR\;=\;\frac{ROI\;\left(HU\right)\;-\;Background\;\left(HU\right)}{\;Background\;SD\;\left(HU\right)}$$

### 2.8. Statistical Analysis

All statistical analyses were performed using Python (version 3.11; Python Software Foundation, Wilmington, DE, USA; python.org) with the SciPy (version 1.13.1; SciPy Developers; scipy.org) and Statsmodels (version 0.14.2; Statsmodels Developers; statsmodels.org) packages. Figures were generated using Matplotlib (version 3.9.2; Matplotlib Development Team; matplotlib.org) and Seaborn (version 0.13.2; Seaborn Developers; seaborn.pydata.org) Normality of the data was assessed using the Shapiro–Wilk test. Because enhancement and CNR measurements were not normally distributed, these data are summarized as medians and interquartile ranges (IQRs).

Differences in enhancement and CNR relative to the reference were calculated at the patient level as the value obtained with each timing protocol minus the corresponding reference value. Friedman tests were used to compare these differences across timing protocols. When significant, post hoc pairwise comparisons between the physiologically derived PTD and each fixed-delay protocol were performed using Wilcoxon signed-rank tests, with *p*-values adjusted for multiple comparisons using the Holm step-down method. A two-sided adjusted *p*-value < 0.05 was considered statistically significant.

Median paired differences relative to the reference were calculated for each timing protocol with 95% confidence intervals estimated using nonparametric bootstrap re-sampling with 10,000 iterations. Pearson’s correlation coefficients (r) were calculated to assess the relationship between reference and observed enhancement and CNR measurements in the MPA for the patient-specific and fixed-delay protocols. Bland–Altman analysis was performed to evaluate agreement, with calculated limits of agreement (LoA) compared with a 10% clinical threshold defined a priori. Because correlation quantifies the strength of association but does not establish agreement between two methods, Bland–Altman analysis was used to quantify systematic bias and the limits of agreement between the predicted and reference measurements. To assess prediction accuracy, the coefficient of determination (R^2^) and root mean square error (RMSE) were also calculated.

Power analysis was performed using pooled effect sizes calculated from paired differences between the patient-specific and fixed-delay protocols across all ROIs. Cohen’s d was used to estimate effect sizes, and the corresponding sample size requirement was determined to achieve 80% power at a significance level of 0.05. Inter-reader reliability was assessed using the intraclass correlation coefficient (ICC) and Spearman’s correlation. Summary enhancement and CNR measurements for each protocol and ROI are presented as median [IQR] in Table 1. Median paired differences relative to the reference and corresponding 95% confidence intervals are presented in Table 2. Mean ± standard deviation values are provided in Supplementary Table S1.

## 3. Results

### 3.1. Study Cohort and Timing Performance

A total of 48 patients who underwent dynamic pulmonary CT perfusion imaging were initially identified. Nine patients were excluded because of excessive motion artifact, inadequate contrast opacification, or field-of-view limitations, resulting in a final study cohort of 39 patients.

The mean physiologically derived PTD was 5.06 s, compared with a mean reference PTD of 5.18 s derived from gamma-variate analysis of the pulmonary arterial enhancement curves. The resulting mean PTD difference between the physiologically derived PTD and the reference timing estimate was 0.12 s across all evaluated vascular territories.

### 3.2. Enhancement and Contrast-to-Noise Ratio Analysis

Enhancement and CNR measurements varied across the evaluated timing protocols, with median [IQR] values for the MPA and lobar pulmonary arteries summarized in Table 1. Median paired differences between each timing protocol and the reference, together with corresponding 95% confidence intervals, are presented in Table 2.

**Figure 3:**
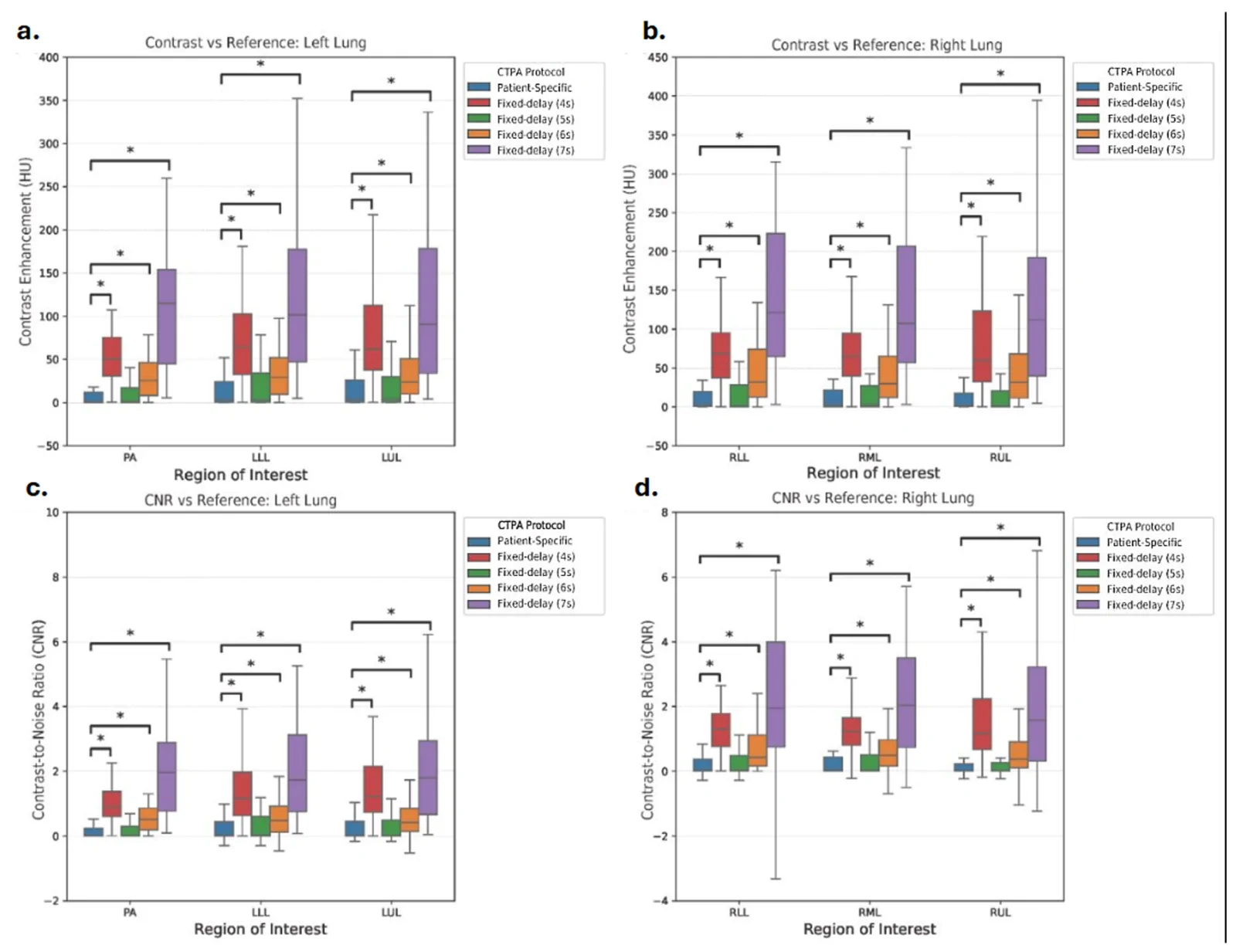
Boxplots showing the distribution of patient-level differences in contrast enhancement and CNR relative to the reference across the MPA and lobar pulmonary arteries for the patient-specific and fixed-delay CTPA protocols. Contrast enhancement differences (**a**,**b**) and CNR differences (**c**,**d**) are shown for the MPA, LLL, LUL, RUL, RML, and RLL. Asterisks (*) indicate statistically significant differences compared with the patient-specific protocol following Wilcoxon signed-rank testing with Holm correction (adjusted *p* < 0.05).

The distribution of patient-level differences from the reference is shown in Figure 3. The physiologically derived PTD demonstrated smaller differences from the reference than the 4-, 6-, and 7-s fixed-delay protocols across all evaluated pulmonary arterial regions, with statistically significant differences following Holm-adjusted Wilcoxon signed-rank testing (*p* < 0.05). No significant differences were observed between the physiologically derived PTD and the 5-s fixed-delay protocol; in the MPA, *p*-values were 0.22 for enhancement and 0.14 for CNR.

**Figure 4:**
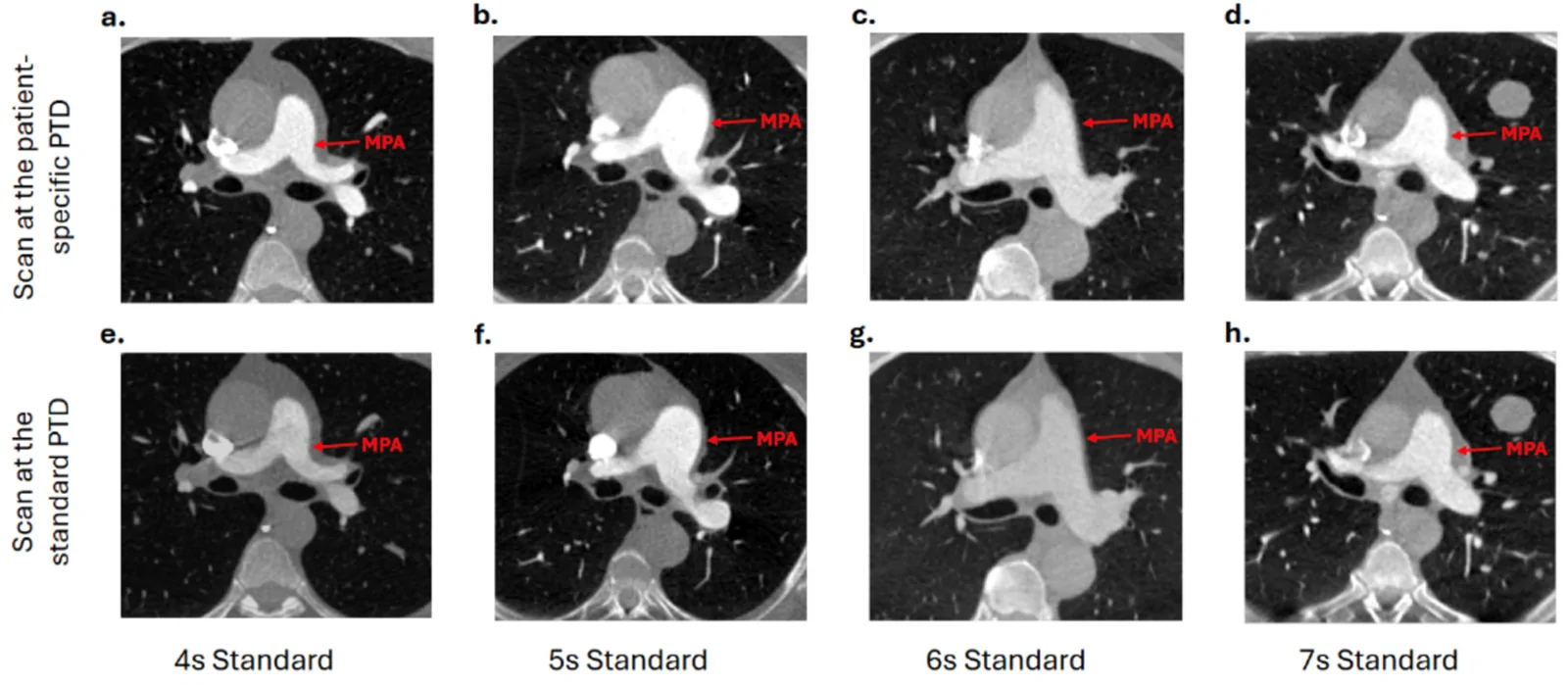
Axial CTPA images at the level of the MPA in four patients (red arrows). The top row (**a**–**d**) shows images acquired using the patient-specific PTD. The bottom row (**e**–**h**) shows corresponding images from the same patients acquired using fixed-scan delays of 4, 5, 6, and 7 s, respectively. CTPA: computed tomography pulmonary angiography; MPA: main pulmonary artery; PTD: post-trigger delay.

Representative qualitative comparisons between the physiologically derived PTD and fixed-delay protocols are shown in Figure 4.

### 3.3. Regression and Agreement Analysis

**Figure 5:**
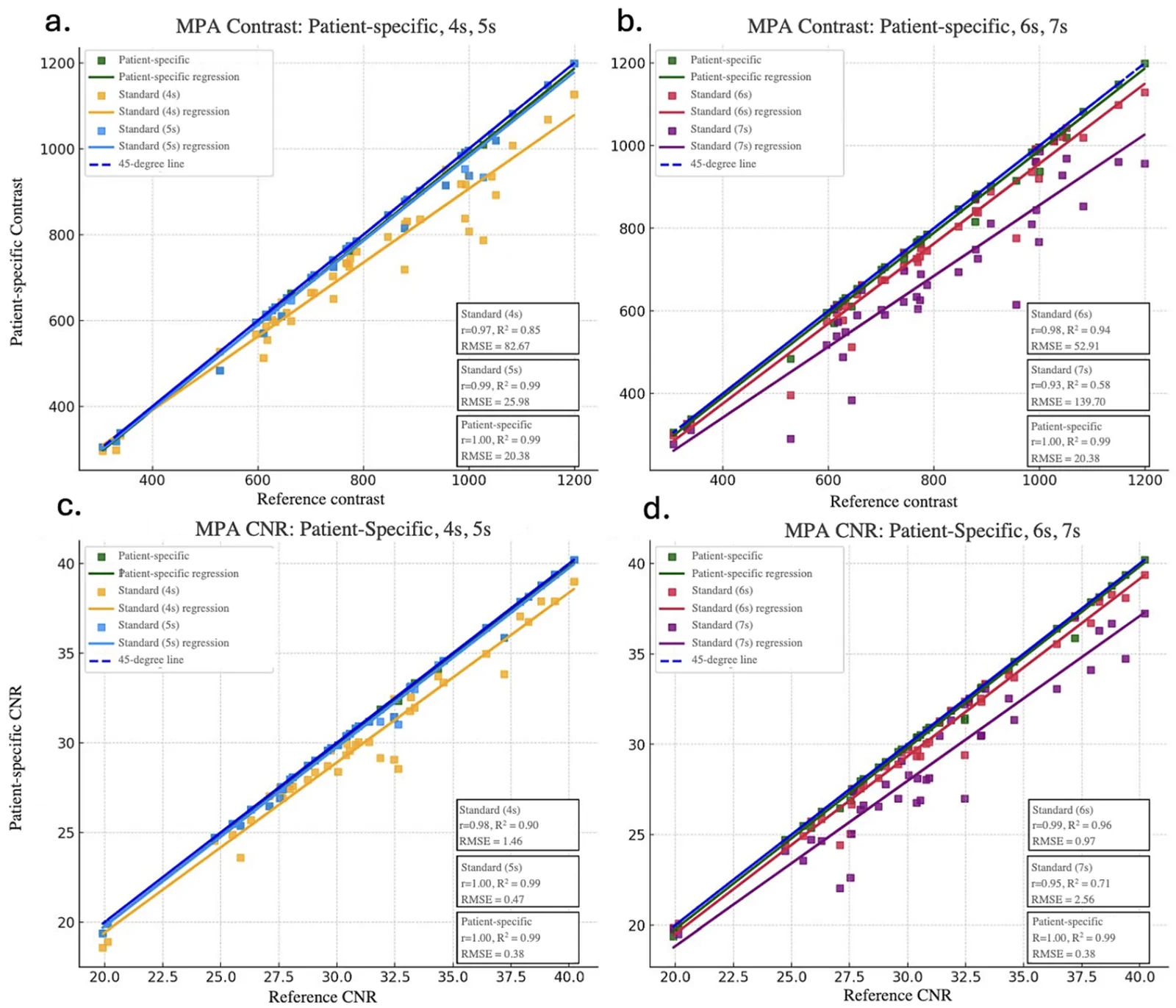
Regression analysis comparing patient-specific and fixed-delay (4–7 s) protocols to reference MPA values for contrast (**a**,**b**) and CNR (**c**,**d**), with regression lines and performance metrics (r, R^2^, RMSE) shown for each protocol. MPA: main pulmonary artery; CNR: contrast-to-noise ratio; r: correlation; R^2^: coefficient of determination; RMSE: root mean square error.

Regression analysis demonstrated strong agreement between physiologically derived PTD measurements and the reference standard in the MPA. For contrast enhancement, the physiologically derived PTD demonstrated the strongest agreement with reference values (r = 1.00, R^2^ = 0.99, RMSE = 20.38), followed by the 5-s, 6-s, 4-s, and 7-s fixed-delay protocols (Figure 5a,b).

Similarly, for CNR, the physiologically derived PTD demonstrated the strongest agreement with reference values (r = 1.00, R^2^ = 0.99, RMSE = 0.38), exceeding the performance observed for all fixed-delay protocols (Figure 5c,d). Detailed regression metrics are summarized in Table 3.

**Figure 6:**
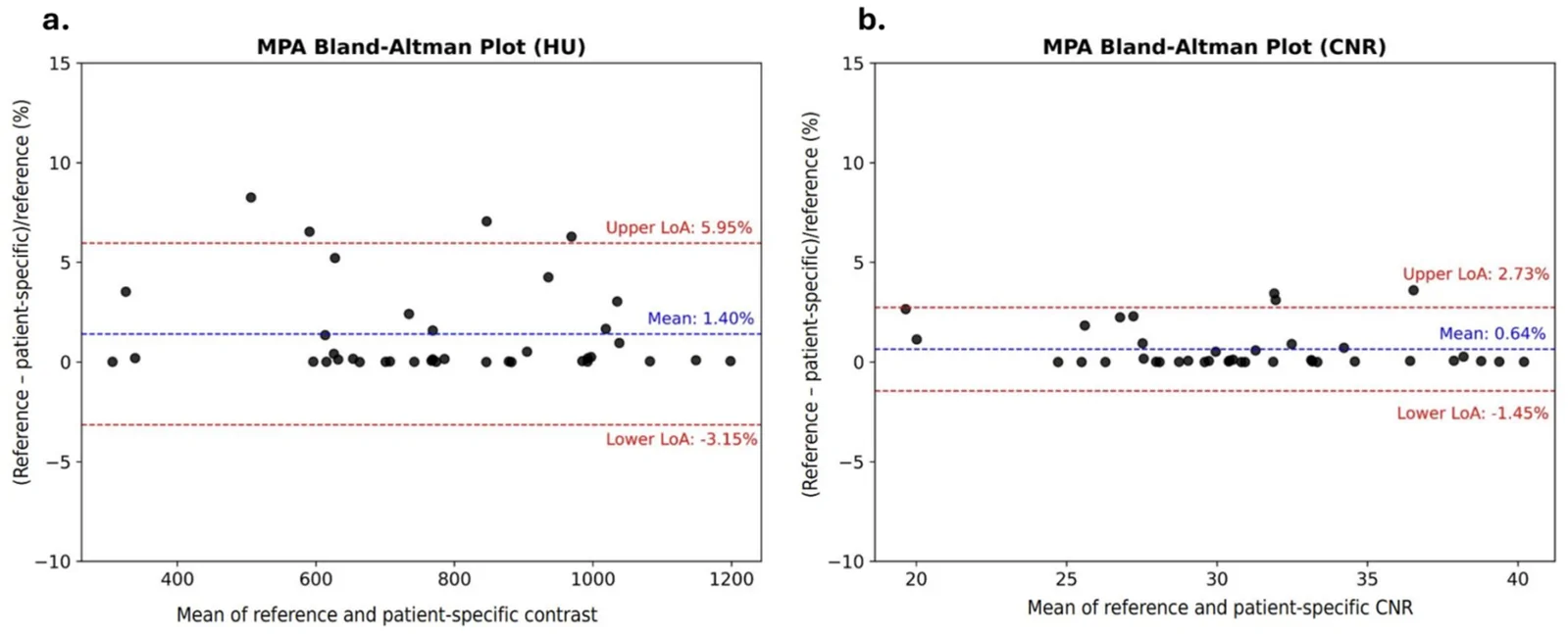
Bland-Altman plots show the percent difference between patient-specific and reference values in the MPA for contrast (**a**) and CNR (**b**), with mean difference and 95% limits of agreement indicated. MPA: main pulmonary artery; CNR: contrast-to-noise ratio.

Bland–Altman analysis demonstrated strong agreement between the physiologically derived PTD and the reference standard for both enhancement and CNR measurements in the MPA (Figure 6). The mean relative difference was 1.40% for enhancement and 0.64% for CNR. The 95% limits of agreement ranged from −3.15% to 5.95% for enhancement and from −1.45% to 2.73% for CNR, both of which fell within the predefined +10% clinical acceptability threshold.

A total of four patients fell outside the limits of agreement for enhancement measurements, while three patients fell outside the limits of agreement for CNR measurements, supporting the overall agreement between physiologically derived PTD estimates and the reference standard.

### 3.4. Effect Size and Power Analysis

Cohen’s d effect sizes were calculated using paired differences in enhancement and CNR error between the physiologically derived PTD and fixed-delay protocols across all pulmonary arterial territories.

For enhancement error in the MPA, effect sizes were 1.07 for the 4-s PTD, 0.22 for the 5-s PTD, 0.67 for the 6-s PTD, and 1.22 for the 7-s PTD. Corresponding CNR effect sizes were 1.11, 0.23, 0.72, and 1.20, respectively.

The pooled effect size across all vascular territories was 0.50 for enhancement error and 0.58 for CNR error, representing moderate-to-large effects. Based on these pooled effect sizes, a minimum sample size of 25 subjects would be required to achieve 80% statistical power at α = 0.05. Therefore, the study cohort of 39 patients exceeded the estimated sample size requirement for detecting meaningful differences in physiologically derived PTD performance.

### 3.5. Interobserver Agreement

ROI placement and image analysis were independently performed by three blinded observers. Interobserver agreement was high, with intraclass correlation coefficients ranging from 0.74 to 0.97 and Spearman correlation coefficients ranging from 0.84 to 0.96 across all measurements (Table 4), indicating strong reproducibility of ROI placement and quantitative image analysis.

## 4. Discussion

The present study evaluated the performance of a physiologically derived PTD model using human dynamic CT perfusion data. The principal finding was that physiologically derived PTD estimates demonstrated strong agreement with retrospectively determined peak pulmonary arterial enhancement timing and consistently produced enhancement and contrast-to-noise ratio (CNR) measurements that closely approximated the reference standard. Furthermore, physiologically derived PTD estimates demonstrated lower enhancement and CNR difference compared to the 4-, 6-, and 7-s fixed-delay protocols and performed similarly to the 5-s fixed-delay protocol.

Unlike conventional fixed-delay approaches, the physiologically derived PTD evaluated in this study was based on a previously established relationship between contrast injection duration and first-pass contrast transport dynamics [24,25,26]. Prior investigations demonstrated that, for relatively short injections, peak enhancement occurs near the temporal center of the dispersed contrast bolus and can be approximated by one-half of the injection duration plus a dispersion component accounting for residual transport and mixing effects within the circulation [24,25,26]. Experimental validation studies further demonstrated that this relationship remained robust across varying hemodynamic conditions and contrast injection protocols [24,25,26]. The present study extends these observations by demonstrating that PTD estimates generated from this previously established physiologic relationship remain consistent with observed pulmonary arterial enhancement timing in human dynamic CT datasets.

The objective of the present study was to evaluate the applicability of a previously established physiologic timing relationship within the human pulmonary circulation. Dynamic CT perfusion imaging provided a unique opportunity to retrospectively characterize patient-specific pulmonary arterial enhancement curves and determine reference peak enhancement timing using gamma-variate modeling. Comparison of physiologically derived PTD estimates with these reference enhancement curves demonstrated close agreement between model-predicted and observed peak enhancement timing.

A notable finding was the similarity in performance between the physiologically derived PTD and the fixed 5-s delay protocol. This likely reflects the relatively narrow distribution of contrast injection durations in the present study; because injection volumes and rates were highly standardized, predicted PTDs clustered near 5 s for most patients. Consequently, a fixed 5-s delay may provide adequate timing under similarly standardized acquisition conditions. The potential advantage of a physiologically derived PTD may become more apparent when contrast bolus geometry or cardiopulmonary transit varies substantially between patients, including differences in iodine delivery rate or contrast concentration [10,13,16,17,18,19,29]. These factors can alter the magnitude, duration, and/or timing of pulmonary arterial enhancement and may therefore reduce the reliability of a single fixed PTD. Importantly, these more heterogeneous clinical conditions were not directly evaluated in the present study, and prospective investigation will be necessary to determine whether physiologically derived timing provides an advantage over a fixed 5-s delay in these settings.

Pulmonary arterial enhancement is influenced by numerous factors, including the contrast injection protocol, cardiac output, circulating blood volume, vascular transit characteristics, and contrast dispersion throughout the cardiopulmonary circulation [4,6,8,9,10,12]. Bolus tracking accounts for interpatient variability in contrast arrival by synchronizing image acquisition with detection of the contrast bolus in the pulmonary circulation. The physiologically derived PTD extends this approach by accounting for injection duration when estimating the interval between bolus detection and peak pulmonary arterial enhancement. Thus, rather than replacing bolus tracking, the PTD model is intended to individualize the post-trigger interval based on the temporal characteristics of the ad-ministered contrast bolus. Although the present study does not evaluate PTD performance across a wide range of injection conditions, the observed agreement with reference peak enhancement supports the underlying physiologic timing relationship under the conditions studied.

Several limitations should be acknowledged. First, this was a retrospective validation study performed at a single institution in a relatively small cohort. The physiologically derived PTD was not prospectively implemented during clinical CTPA acquisition; rather, its performance was evaluated against peak enhancement timing retrospectively derived from dynamic CT data using gamma-variate modeling. Accordingly, agreement between the predicted PTD and reference enhancement timing validates the physiologic timing relationship but does not establish that implementation of the model improves diagnostic image quality, diagnostic accuracy, or clinical outcomes. Prospective implementation will be necessary to determine whether improved prediction of enhancement timing translates into meaningful clinical benefit.

Second, all examinations were performed using a single scanner platform and a highly standardized contrast administration protocol, resulting in a relatively narrow range of injection durations and limiting generalizability to more heterogeneous clinical protocols. Although the underlying relationship between injection duration and first-pass contrast enhancement is physiologically based, its performance may be influenced by scanner-dependent acquisition delays, iodine concentration and delivery rate, and differences in temporal resolution. Emerging technologies such as photon-counting CT may further alter acquisition characteristics and contrast requirements [30,31]. Therefore, the stability of the proposed PTD relationship should be evaluated across scanner vendors, reconstruction methods, contrast protocols, and newer CT technologies before broader clinical implementation. Finally, the study population was not representative of the population in which CTPA is most commonly performed for suspected pulmonary embolism. Most examinations were performed for pulmonary lesion assessment, and only one patient in the study cohort had pulmonary embolism. Thus, the present findings primarily evaluate prediction of pulmonary arterial enhancement timing rather than diagnostic performance for pulmonary embolism.

Future investigations should prospectively evaluate the physiologically derived PTD across broader ranges of injection duration, injection rate, iodine delivery rate, scanner platforms, and patient populations, particularly those with obesity, impaired cardiac function, or pulmonary hypertension [16,17,32]. Such studies should determine whether the timing relationship remains stable under heterogeneous clinical conditions and, importantly, whether prospective implementation improves pulmonary arterial enhancement, di-agnostic image quality, or diagnostic performance compared with conventional fixed-delay protocols.

The findings of this study should also be interpreted within the context of previous investigations evaluating contrast timing strategies for CT angiography. Traditional test-bolus and bolus-tracking approaches attempt to account for variability in contrast arrival time but typically rely on empirically selected delay intervals following triggering [6,21,33]. In contrast, the physiologically derived PTD model seeks to estimate the interval between triggering and peak enhancement using a relationship derived from first-pass contrast transport dynamics [24,25,26]. The strong agreement observed between physiologically derived PTD estimates and retrospectively determined peak enhancement timing suggests that fundamental characteristics of contrast transport may provide a useful framework for predicting enhancement timing within the pulmonary circulation.

## 5. Conclusions

In conclusion, the physiologically derived PTD demonstrated strong agreement with reference pulmonary arterial peak enhancement timing in this human cohort. Enhancement and CNR values were comparable to the reference standard and showed smaller deviations than fixed delays of 4, 6, and 7 s, while performing similarly to a 5-s fixed delay under the standardized injection conditions evaluated. These findings validate a previously established physiologic PTD relationship using human dynamic CT data and support further prospective evaluation across diverse contrast injection protocols, scanner platforms, and patient populations. However, this study assessed enhancement timing prediction only and did not evaluate diagnostic performance for pulmonary embolism or clinical outcomes.

## Figures and Tables

**Table 1:** Pulmonary arterial enhancement and contrast-to-noise ratio across timing protocols. Enhancement (HU) and contrast-to-noise ratio (CNR) measurements are presented as median [interquartile range] for the reference, physiologically derived PTD, and fixed-delay protocols across the main pulmonary artery and lobar pulmonary arteries. Asterisks (*) indicate statistically significant differences compared with the physiologically derived PTD following Wilcoxon signed-rank testing with Holm adjustment for multiple comparisons (adjusted *p* < 0.05). MPA, main pulmonary artery; LLL, left lower lobar artery; LUL, left upper lobar artery; RLL, right lower lobar artery; RML, right middle lobar artery; RUL, right upper lobar artery; HU, Hounsfield units; CNR, contrast-to-noise ratio; PTD, post-trigger delay; IQR, interquartile range.

ROI		Reference	Patient-Specific	4 s Standard	5 s Standard	6 s Standard	7 s Standard
MPA	HU Median [IQR]: CNR Median CNR [IQR]:	773.42 [638.23–988.36] 30.55 [27.81–33.25]	768.15 [628.06–960.81] 30.51 [27.75–33.25]	732.44 [600.76–836.86] 29.31 [27.47–32.49] *	773.39 [628.06–935.19] 30.51 [27.75–33.14]	739.88 [612.89–928.14] 29.67 [27.56–32.94]	650.60 [581.74–827.85] 28.12 [26.47–32.37]
LLL	HU Median [IQR]: CNR Median CNR [IQR]:	848.51 [657.07–951.85] 29.62 [27.66–33.14]	816.84 [659.05–929.98] 29.32 [27.49–32.82]	739.66 [607.25–853.43] 28.46 [26.39–31.64]	805.70 [659.05–928.98] 29.32 [27.49–32.82]	840.97 [685.45–933.92] 29.32 [27.18–32.32]	734.02 [609.30–864.66] 28.41 [25.94–31.14]
LUL	HU Median [IQR]: CNR Median CNR [IQR]:	739.00 [604.56–932.43] 30.13 [26.27–32.49]	738.75 [604.60–901.93] 29.39 [25.98–32.43]	671.47 [557.69–813.86] 27.33 [24.95–31.31] *	738.75 [600.32–890.89] 29.39 [25.98–32.57]	718.86 [611.45–908.27] 30.09 [25.73–32.35]	638.26 [540.80–833.00] 28.59 [24.47–30.95]
RLL	HU Median [IQR]: CNR Median CNR [IQR]:	844.25 [682.04–988.87] 330.79 [27.85–33.28]	816.49 [656.69–971.96] 30.36 [27.78–33.08]	731.71 [571.78–864.83] 28.63 [26.26–31.95]	816.49 [656.69–951.77] 30.34 [27.56–33.28]	804.00 [634.19–915.85] 29.98 [27.18–32.88]	706.59 [573.72–797.94] 28.49 [26.12–30.87]
RML	HU Median [IQR]: CNR Median CNR [IQR]:	775.03 [574.53–943.27] 29.49 [27.15–34.21]	728.99 [576.49–925.46] 28.99 [26.73–33.16]	617.28 [507.01–825.71] 27.72 [25.09–32.20] *	744.17 [576.49–925.46] 28.99 [26.73–33.16]	730.43 [570.99–896.89] 29.28 [26.70–33.72] *	648.39 [501.65–813.34] 27.95 [24.70–31.23]
RUL	HU Median [IQR]: CNR Median CNR [IQR]:	737.20 [592.43–925.60] 30.06 [27.75–33.08]	727.32 [586.99–883.27] 29.56 [27.34–32.89]	659.33 [532.63–851.65] 29.07 [25.43–32.46]	727.32 [586.99–883.27] 29.56 [27.34–32.89]	692.73 [548.67–867.80] 29.57 [26.78–33.11]	646.36 [487.17–771.29] 27.78 [25.61–31.13]

**Table 2:** Absolute differences in pulmonary arterial enhancement and contrast-to-noise ratio relative to the reference. Absolute differences in enhancement (HU) and contrast-to-noise ratio (CNR) relative to the reference are presented as median (95% confidence interval) for the physiologically derived PTD and fixed-delay protocols across the main pulmonary artery and lobar pulmonary arteries. Confidence intervals were estimated using nonparametric bootstrap resampling with 10,000 iterations. Asterisks (*) indicate statistically significant differences compared with the physiologically derived PTD following Wilcoxon signed-rank testing with Holm adjustment for multiple comparisons (adjusted *p* < 0.05). MPA, main pulmonary artery; LLL, left lower lobar artery; LUL, left upper lobar artery; RLL, right lower lobar artery; RML, right middle lobar artery; RUL, right upper lobar artery; HU, Hounsfield units; CNR, contrast-to-noise ratio; PTD, post-trigger delay; CI, confidence interval.

ROI		Patient-Specific	4 s Standard	5 s Standard	6 s Standard	7 s Standard
MPA	Absolute HU difference vs. Reference, median (95% CI) Absolute HU difference vs. Reference, median (95% CI)	1.02 (0.42–4.66) 0.02 (0.01–0.10)	50.76 (35.53–66.88) * 0.89 (0.67–1.23) *	1.05 (0.48–8.35) 0.02 (0.01–0.16)	25.60 (17.95–40.34) * 0.51 (0.32–0.78) *	114.66 (78.45–138.55) * 1.96 (1.46–2.67) *
LLL	Absolute HU difference vs. Reference, median (95% CI) Absolute HU difference vs. Reference, median (95% CI)	2.87 (0.74–11.24) 0.04 (0.02–0.28)	64.50 (40.58–82.95) * 1.15 (0.76–1.52) *	2.87 (0.74–16.88) 0.04 (0.02–0.32)	29.25 (15.63–43.93) * 0.51 (0.28–0.79) *	101.49 (63.44–134.95) * 1.84 (1.12–2.82)*
LUL	Absolute HU difference vs. Reference, median (95% CI) Absolute HU difference vs. Reference, median (95% CI)	3.99 (0.68–10.39) 0.06 (0.02–0.20)	62.01 (45.33–80.17) * 1.22 (0.83–1.51) *	3.99 (0.68–14.92) 0.06 (0.02–0.30)	23.75 (13.53–40.44) * 0.49 (0.29–0.69) *	90.92 (62.66–141.94) * 1.83 (1.25–2.73) *
RLL	Absolute HU difference vs. Reference, median (95% CI) Absolute HU difference vs. Reference, median (95% CI)	3.35 (0.77–11.85) 0.03 (0.01–0.28)	69.14 (49.70–89.38) * 1.30 (0.98–1.72) *	1.49 (0.55–11.85) 0.03 (0.01–0.28)	31.70 (19.80–55.96) * 0.68 (0.33–1.05)*	121.50 (83.24–178.89) * 2.25 (1.45–3.45) *
RML	Absolute HU difference vs. Reference, median (95% CI) Absolute HU difference vs. Reference, median (95% CI)	2.99 (0.43–11.04) 0.04 (0.01–0.23)	65.25 (50.99–84.16) * 1.24 (0.97–1.48) *	2.99 (0.43–17.13) 0.04 (0.01–0.34)	29.72 (16.40–51.57) * 0.65 (0.41–0.95) *	107.55 (87.09–172.49) * 2.15 (1.45–3.41) *
RUL	Absolute HU difference vs. Reference, median (95% CI) Absolute HU difference vs. Reference, median (95% CI)	1.57 (0.86–8.05) 0.06 (0.02–0.22)	60.08 (39.06–79.58) * 1.17 (0.83–1.60) *	1.39 (0.80–8.05) 0.04 (0.01–0.23)	31.25 (17.89–52.33) * 0.62 (0.37–0.99) *	112.09 (67.36–172.09) * 2.19 (1.20–3.16) *

**Table 3:** r, R^2^, and RMSE between reference values for each protocol for contrast and CNR in the MPA. r: correlation; R^2^: coefficient of determination; RMSE: root mean square error; CNR: contrast-to-noise ratio; MPA: main pulmonary artery.

MPA		Reference vs. Patient-Specific	Reference vs. 4 s Standard	Reference vs. 5 s Standard	Reference vs. 6 s Standard	Reference vs. 7s Standard
Contrast	r	1.00	0.97	0.99	0.98	0.93
	R^2^	0.99	0.85	0.99	0.94	0.58
	RMSE	20.38	82.67	25.98	52.91	139.70
CNR	r	1.00	0.98	1.00	0.99	0.95
	R^2^	0.99	0.90	0.99	0.96	0.71
	RMSE	0.38	1.46	0.47	0.97	2.56

**Table 4:** Inter-observer agreement measured using the ICC and Spearman correlation coefficients. ICC: intraclass correlation coefficient.

Observer Pair	ICC	Spearman
Observer 1 vs. Observer 2	0.742	0.844
Observer 1 vs. Observer 3	0.758	0.839
Observer 2 vs. Observer 3	0.972	0.964

## Data Availability

The data supporting the findings of this study are subject to institutional data use restrictions. Deidentified CT imaging datasets were obtained under a data use agreement and are not publicly available due to participant privacy and institutional policy. Derived quantitative imaging data are available from the corresponding author upon reasonable request.

## Supplementary Materials

The following supporting information can be downloaded at: https://www.mdpi.com/article/10.3390/diagnostics16183033/s1, Table S1: Mean pulmonary arterial enhancement and contrast-to-noise ratio across timing protocols. Demographics information is included in the supplementary materials.

## Abbreviations

The following abbreviations are used in this manuscript:

AIF	Arterial Input Function
CNR	Contrast-to-noise ratio
CTPA	Computed tomography pulmonary angiography
HU	Hounsfield units
ICC	Intraclass Correlation Coefficient
LLL	Left lower lobe
LUL	Left upper lobe
MPA	Main pulmonary artery
PTD	Post-trigger delay
R^2^	Coefficient of determination
RLL	Right lower lobe
RML	Right middle lobe
ROI	Region of interest
RUL	Right upper lobe
RMSE	Root Mean square error
TTP	Time to peak enhancement
