# Supplementary material for: Patient-Specific Timing for Pulmonary CT Angiography: Human Validation of a Physiologically Derived Post-Trigger Delay Model
Source: Diagnostics (Basel). 2026 Sep 19;16(18):3033. doi: 10.3390/diagnostics16183033 (PMC13605558; doi:10.3390/diagnostics16183033)
Supplement: Supplementary file 1 [file diagnostics-16-03033-s001.zip › diagnostics-4467527-supplementary.pdf]

	ROI	Reference	Patient-Specific	4s Standard	5s Standard	6s Standard	7s standard
**MPA**	Contrast:<br>CNR:<br>Difference (HU, CNR)	789.58 ± 218.87<br>30.92 ± 4.79	779.03 ± 218.35<br>30.73 ± 4.81<br>(-10.55, -0.19)	726.15±193.28<br>29.78 ± 4.62<br>(-63.43, -1.14) *	775.97± 215.85<br>30.68 ± 4.80<br>(-13.61, -0.24)	752.89 ± 215.24<br>30.24 ± 4.75<br>(-36.69, -0.68) *	675.73 ± 202.25<br>28.81 ± 4.59<br>(-113.85, -2.11) *
**LLL**	HU:<br>CNR:<br>Error (HU, CNR)	812.25 ± 220.04<br>30.42 ± 4.37	797.57 ± 220.38<br>30.15 ± 4.37<br>(-14.68, -0.27)	734.08 ± 205.56<br>29.01 ± 4.31<br>(-78.17, -1.41) *	794.80 ± 219.55<br>30.10 ± 4.36<br>(-17.45, -0.32)	780.22 ± 213.15<br>29.81 ± 4.33<br>(-32.03, -0.61) *	702.53 ± 198.39<br>28.44 ± 4.36<br>(-109.72, -1.98) *
**LUL**	HU:<br>CNR:<br>Error (HU, CNR)	751.90 ± 229.12<br>30.01 ± 4.96	737.81 ± 226.44<br>29.76 ± 4.92<br>(-14.09, -0.25)	671.38 ± 203.73<br>28.53 ± 4.52<br>(-80.52, -1.48) *	735.30 ± 224.60<br>29.72 ± 4.92<br>(-16.6, -0.29)	721.25 ± 222.13<br>29.51 ± 5.10<br>(-30.65, -0.5) *	650.12 ± 212.02<br>28.20 ± 5.20<br>(-101.78, -1.81) *
**RLL**	HU:<br>CNR:<br>Error (HU, CNR)	806.74 ± 243.38<br>30.87 ± 4.30	789.52 ± 240.26<br>30.56 ± 4.26<br>(-17.22, -0.31)	715.82 ± 227.89<br>29.21 ± 4.18<br>(-90.21, -1.66) *	786.92 ± 237.58<br>30.52 ± 4.34<br>(-19.82, -0.35)	778.17 ± 230.63<br>30.34 ± 4.25<br>(-28.57, -0.53) *	704.99 ± 227.32<br>28.95 ± 4.23<br>(-101.74, -1.92) *
**RML**	HU:<br>CNR:<br>Error (HU, CNR)	727.05 ± 235.91<br>30.98 ± 4.91	712.70 ± 230.22<br>30.72 ± 4.78<br>(-14.35, -0.26)	636.76 ± 216.19<br>29.26 ± 4.48<br>(-90.29, -1.72) *	709.96 ± 224.58<br>30.67 ± 4.69<br>(-17.09, -0.31)	705.02 ± 224.19<br>30.59 ± 5.04<br>(-22.03, -0.39) *	635.91 ± 225.98<br>29.30 ± 5.50<br>(-91.14, -1.68) *
**RUL**	HU:<br>CNR:<br>Error (HU, CNR)	721.75± 213.00<br>30.82±4.63	711.84±204.19<br>30.64± 4.52<br>(-9.91, -0.18)	634.41± 191.61<br>29.20± 4.55<br>(-87.34, -1.62) *	709.49± 200.99<br>30.60 ± 4.51<br>(-12.26, -0.22)	703.46± 204.08<br>30.49± 4.57<br>(-18.29, -0.33) *	635.19 ± 215.74<br>29.26± 4.99<br>(-86.56, -1.56) *

Supplementary Table S1. Mean pulmonary arterial enhancement and contrast-to-noise ratio across timing protocols. Enhancement (HU) and contrast-to-noise ratio (CNR) measurements are presented as mean ± standard deviation for the reference, physiologically derived PTD, and fixed-delay protocols across the main pulmonary artery and lobar pulmonary arteries. These data are provided as supplementary descriptive statistics; primary comparisons are reported using median-based nonparametric analyses in the main manuscript.
